# Assessing Return to Work Outcomes for Individuals Affected by Burn Injuries: A Comprehensive Study

**DOI:** 10.7759/cureus.54410

**Published:** 2024-02-18

**Authors:** Kush Verma, Sangeeta Thakurani, Aakansha Vashishta, Sriranjani I Srivatsa, Deepti Shah

**Affiliations:** 1 Plastic and Reconstructive Surgery, SMS (Sawai Man Singh) Medical College, Jaipur, IND; 2 Anaesthesiology, SMS (Sawai Man Singh) Medical College, Jaipur, IND

**Keywords:** burn types, employment status, burn rehabilitation, return to work, burn injuries

## Abstract

Background

Burn injuries can have long-lasting effects on individuals, including their ability to return to work (RTW). This study aims to comprehensively analyze factors influencing the RTW status of burn patients after their injuries.

Methods

A dataset containing information on gender, age groups, burn types, discharge status, burn causes, employment status, total body surface area (TBSA) burn, and more were analyzed. The dataset covered the years 2018 to 2020. Chi-square tests were used for categorical data, while Mann-Whitney U tests were used for continuous variables. The participant characteristics, activity impairment, and work results were investigated using descriptive statistics.

Results

The number of reported burn cases was higher among males than females in 2018, 2019, and 2020. The highest burn cases occurred within the 25-40 age group. Most of the patients were involved in manual labor-intensive work prior to burn injury, unemployed individuals also accounted for a notable proportion of the cases. Most patients analyzed for the study had sustained 20-40% TBSA burn. From a total of 1130 patients, 710 (62.83%) of patients returned to work, and (37.16%) did not RTW.

Conclusion

Understanding the factors influencing the RTW status of burn patients after one year is crucial for effective occupational rehabilitation. This analysis provides insights into gender differences, age distribution, burn types, discharge outcomes, causes of burn incidents, employment status, TBSA burn, and the relationship between these factors and RTW rates.

## Introduction

Empirical investigations and theoretical considerations of employment and unemployment support the assumption that labor is helpful for health and well-being. Being able to work, therefore, serves as a crucial component of one's identity, social duties, and social status, as well as a means of generating revenue. Hence, it is essential to define return to work (RTW) in burn patients. We define RTW as being able to perform activities of daily living independently, earning satisfactory employment and income (maintaining or increasing), and in the case of females (who were homemakers before suffering burns), continuing to perform household activities in similar capabilities as before. Being able to RTW after suffering a burn injury is linked to normalcy and social integration, whereas not doing so has been linked to physical impairment and poor health-related quality of life. RTW is, therefore, one of the most crucial goals to pursue in burn recovery [1].

Due to significant advancements in burn care, severely damaged burn sufferers have a high chance of surviving. In Western civilization, there are more burn survivors due to the general death rate being lower. Significant morbidity (such as scarring, contractures, discomfort, psychological stress, and weakness) is more common in patients with severe burns, which makes it difficult for them to return to employment and society. Much work is invested in creating multidisciplinary and early rehabilitation programs for this patient population. RTW has earned attention as a crucial result of burn treatment more recently. Work is an essential source of money for people and is also advantageous for many personal, social, and societal elements [2].

The International Classification of Functioning (ICF) is the fundamental set used to pinpoint important elements and procedures pertinent to a job evaluation, such as specific body composition and functions, activity and participation restrictions, and social and environmental supports for a successful RTW. Age, comorbidity, psychological and psychiatric factors, pre-burn employment status (employed or self-employed), other job-related factors, insurance status, total body surface area (TBSA) of partial (PTB) and full thickness burns (FTBS), burn location (hand and facial involvement), pain, and several treatment characteristics, as a proxy of severity (surgical procedures, the use of anesthesia, etc.), have all been identified as important factors in RTW after burns [3,4]. Most people RTW following a burn; however, recovery after a serious burn might occasionally take a while [5]. Promoting community engagement, including employment, is a key objective of rehabilitation therapy for burn victims. For those who have suffered burn injuries, finding work can be difficult due to various factors, including scars, contractures, discomfort, weakness, amputations, psychological problems, and concerns with appearance. Therefore, while offering a thorough rehabilitation program, it is crucial to comprehend how these concerns affect the capacity to perform after a burn injury and treat these issues [3].

Employment is crucial for burn patients' rehabilitation, as work productivity and capacity to return to their pre-burn jobs. Work productivity may be affected when a person cannot function at a level comparable to that before an accident or must find another profession due to burn sequelae. For burn victims, participation in everyday activities and social life is crucial [6]. Less is known about how burn survivors do financially following injury and if they work in similar fields and occupations. There have not been any studies to determine if burn victims can return to their pre-injury productivity (i.e., earn the same amount of money or output at work). Identifying patients who run the danger of not regaining their pre-injury productivity is crucial to providing these people and their families with the help they need. Even though burn injuries happen most often at home, a multicenter study of people with serious burn injuries revealed that 42% of those working outside the house at the time of the incident had received damage at work [7].

Returning to work after a burn injury can be a challenging and complex process for many individuals. That's why it is essential to understand the key factors that affect the RTW process and to identify the proportion of burn victims who RTW and when. This study aims to provide evidence-based insights into the key domains affecting RTW following a burn injury. By compiling the evidence for each area, we can make informed recommendations and guidelines for future research on RTW after a burn injury. Our goal is to improve the quality of life for burn victims and help them RTW with confidence and dignity.

## Materials and methods

This prospective study was conducted at SMS Medical College and Hospital from January 2018 to December 2020. The database includes burn patients admitted to the intensive care unit (ICU) at the hospital's burn center.

Inclusion criteria

All patients who were admitted to the hospital provided their consent to participate in the experiment, including both male and female patients who were ≥18 years and <60 years, individuals without a history of dementia or mental retardation, >5% of their TBSA burned, or a length of stay (LOS) greater than one day and who were discharged after completion of treatment (surgical/conservative). The database was followed up by a survey at six, 12, and 24 months from discharge. All participants who reported at any subsequent follow-up interval were included.

Exclusion criteria

Patients temporarily admitted to the hospital but who received their primary care elsewhere were excluded.

The study was conducted after the patients met the inclusion and exclusion criteria for the study and after receiving clearance from the institutional ethics committee.

The database was followed up by a survey at six, 12, and 24 months from the day of discharge and asked about activities of daily living and employment status. All participants who reported at any subsequent follow-up interval were included.

Statistical analysis

Chi-square tests were employed for categorical data, while Mann-Whitney U-tests were utilized for continuous variables. Descriptive statistics examined participant characteristics, activity impairment, and work outcomes. The full sample and subgroups based on burn severity were analyzed. The characteristics and results of the two burn severity subgroups were compared. The traits of individuals with and without work were contrasted as well.

## Results

The dataset provided encompasses information regarding burn injuries, including gender, burn types, employment status, TBSA burn, body areas affected, and type of management concerning RTW, which has been summarized in Table 1. Data were collected by a survey for two years.

**Table 1:** Patient's RTW status after burn RTW: return to work; TBSA: total body surface area.

N = 1130	Number of patients (n)		Returned to work		Not returned to work		P-value
Male	790		489		301		0.322, not significant
Female	340		221		119		
Gender & age distribution	M	F	M	F	M	F	
18-25	170	63	56	45	114	18	<0.00001, highly significant
25-40	232	105	156	46	76	59	
40-50	203	92	133	73	70	19	
50-60	185	80	144	57	41	23	
Pre-injury employment status							
Manual work/unskilled/homemakers	508		241		267		<0.00001, highly significant
Desk job/office/service/business	340		282		58		
Unemployed	282		187		95		
TBSA burn							
Less than 20%	339		233		106		0.005419, highly significant
20-40%	380		236		144		
40-60%	230		127		107		
>60%	181		114		63		
Cause of burn injury							
Flame	587		436		151		<0.00001, highly significant
Scald	170		146		24		
Electrical	353		123		230		
Chemical	20		5		15		
Type of management of burn injury							
Conservative	678		460		218		<0.00001, highly significant
Minor surgical (debridement/≤ two-digit amputation)	249		180		69		
Major surgical (major amputation/flap cover)	203		70		133		
Body part involved (more than one can be involved)							
Head & neck	508		304		204		0.001181, highly significant
Limb	791		445		346		
Trunk/buttocks/genitalia	904		587		317		

**Figure 1:**
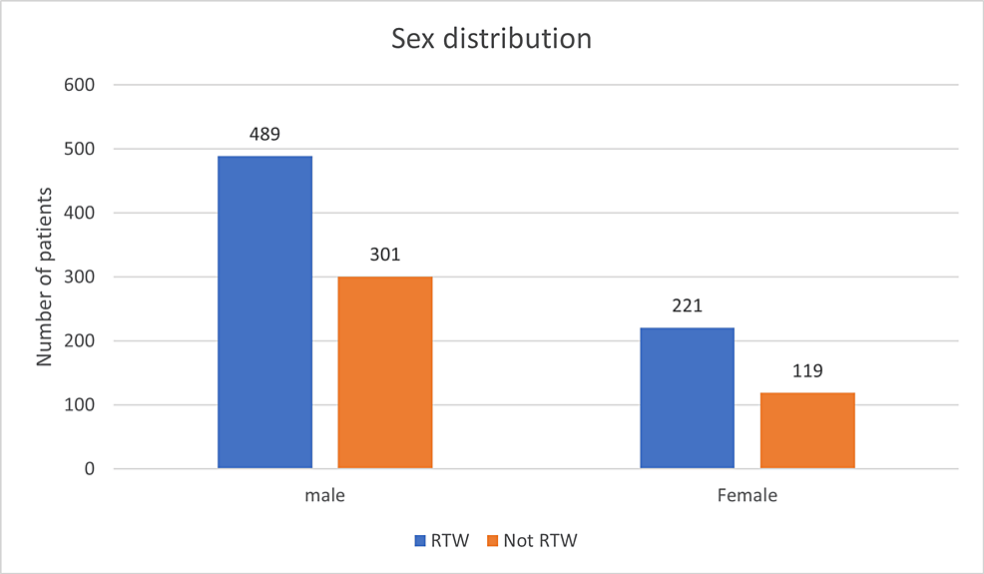
RTW and sex distribution RTW: return to work.

**Figure 2:**
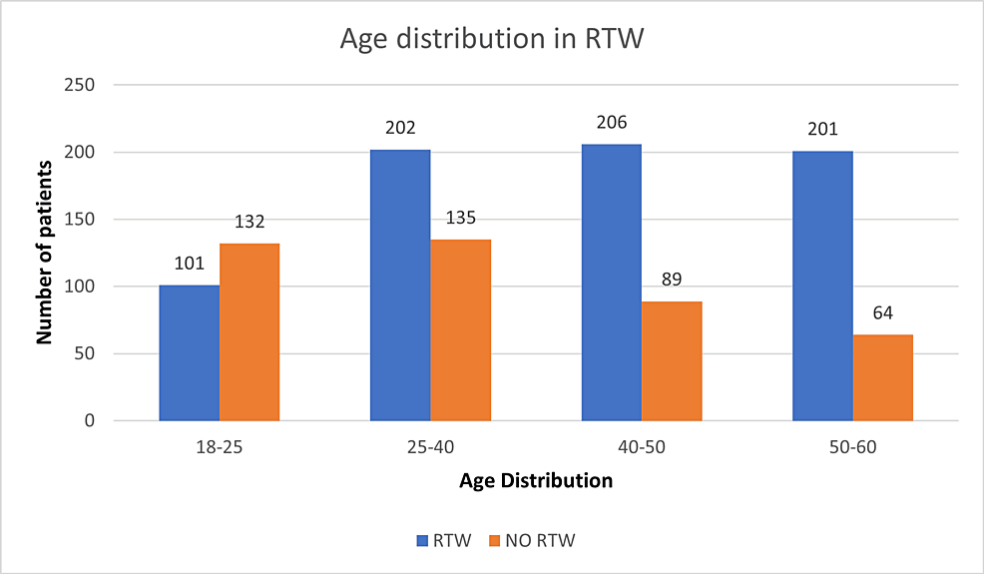
Age distribution and RTW RTW: return to work.

**Figure 3:**
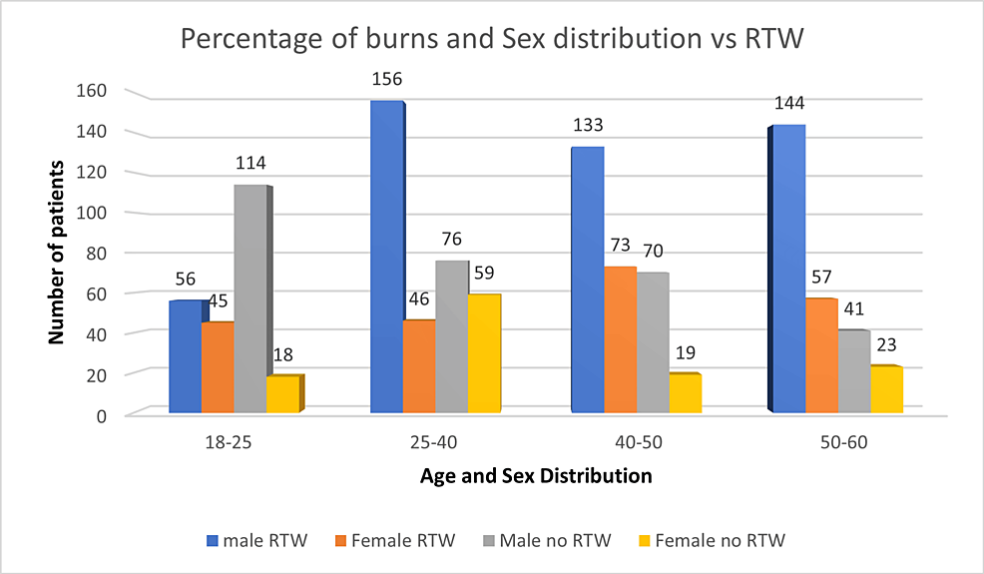
Age and sex bracket distribution and RTW RTW: return to work.

**Figure 4:**
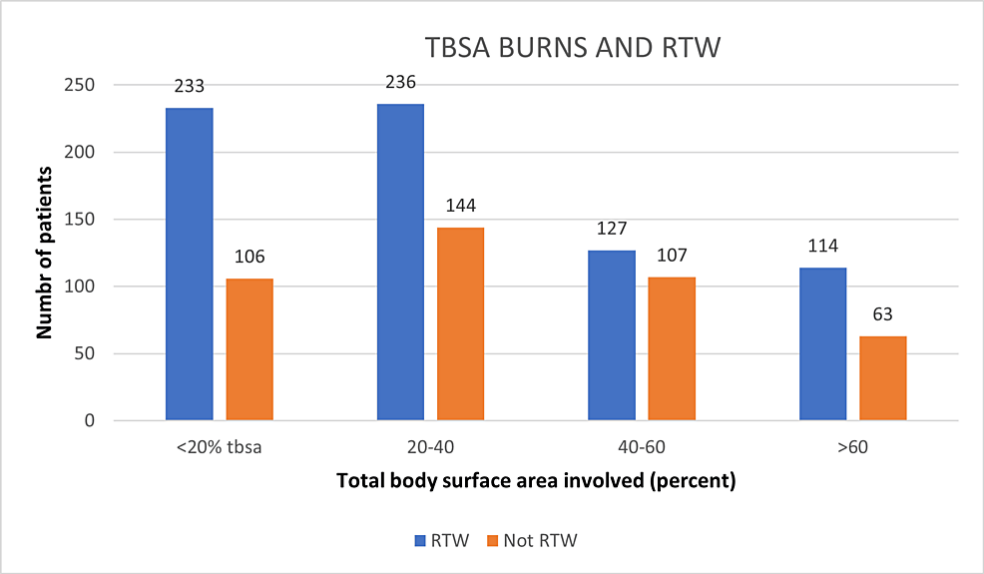
Total body surface area burns and RTW. RTW: return to work; TBSA: total body surface area.

**Figure 5:**
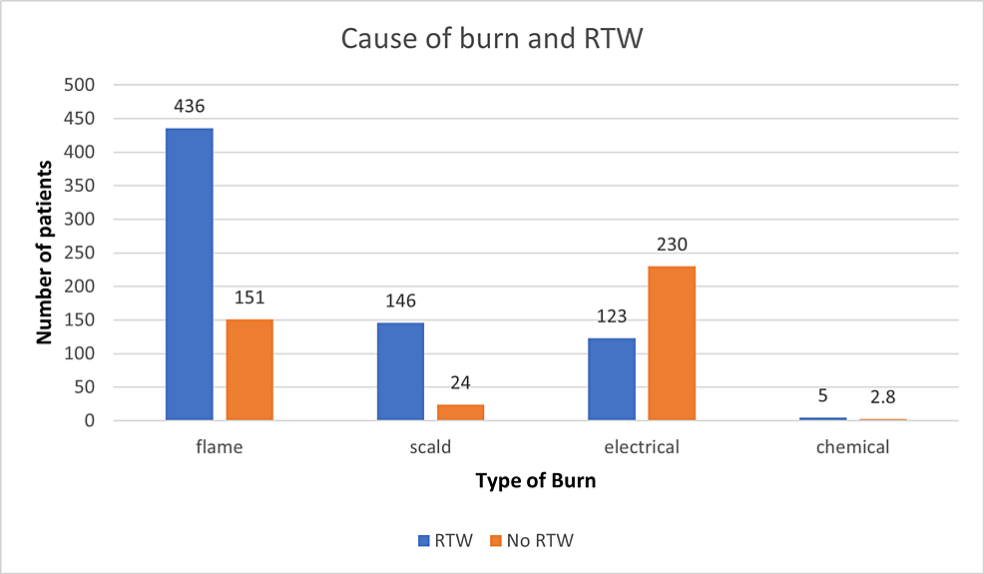
Cause of burn injury and RTW RTW: return to work.

**Figure 6:**
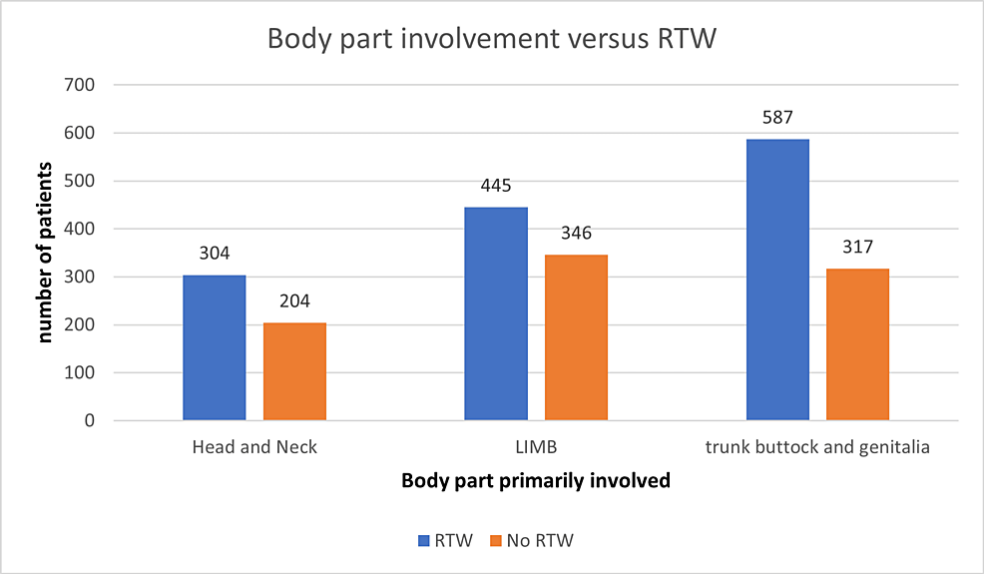
Body part(s) involved primarily and RTW RTW: return to work.

**Figure 7:**
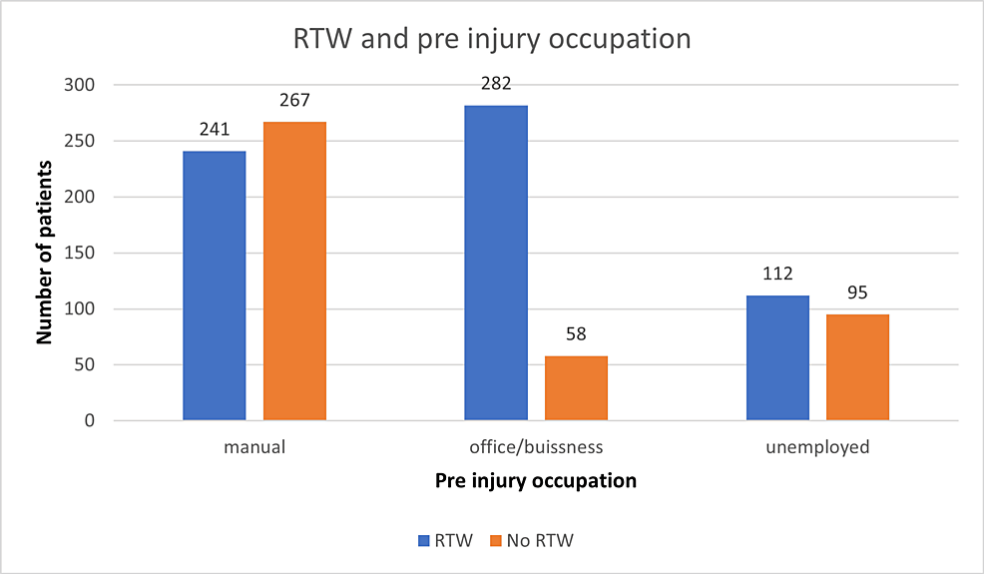
Preinjury occupation status and RTW RTW: return to work.

**Figure 8:**
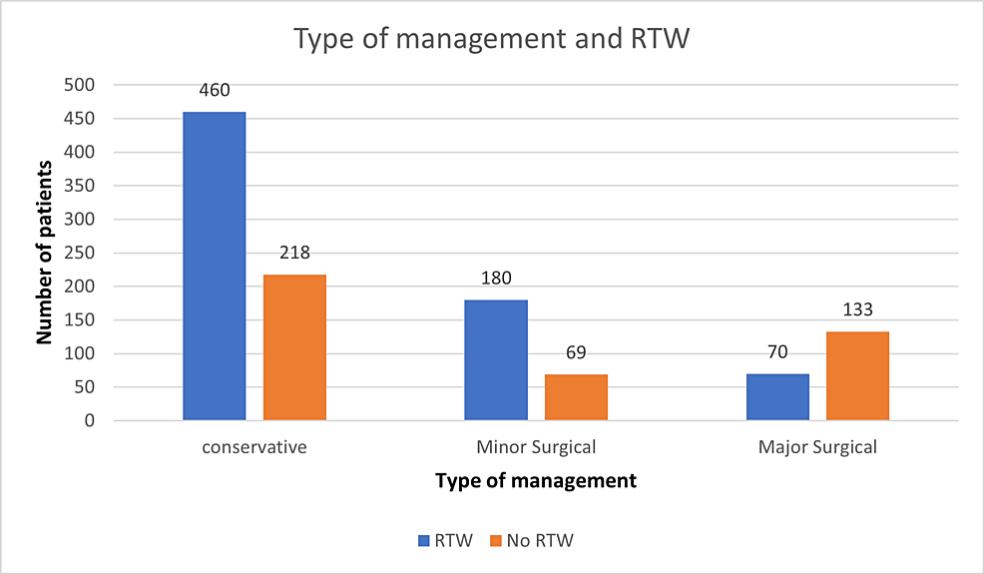
Type of burn injury management provided and RTW RTW: return to work.

A total of 1130 patients were followed up in this study, including 790 males and 340 females, of which 489 and 221 patients returned to work, but the difference was insignificant (Figure 1). Overall, the age distribution pattern showed that younger patients <25 years had poor RTW outcomes (Figure 2). When this subset of data was further studied with gender-specific age distributions, it was found that young males who were less than 25 years old had poor RTW, and females who were in the 25-40 years age group had poor RTW (Figure 3). The TBSA affected by burns varied across the cases. Most burn cases reported TBSA percentages of less than 20% and 20-40%. However, fewer cases exhibited higher TBSA percentages, indicating severe burns. A higher percentage of burns had poorer RTW outcomes (Figure 4). The causes of burn injuries were categorized into flame, scald, electrical, and chemical. Flame burns were the most common cause, followed by electrical burns. Scald burns accounted for a smaller proportion, and chemical burns were relatively rare. It was observed that electric burns, which are usually deep and associated with limb involvement, had poorer RTW while those with other thermal burns had better RTW with results being statistically significant (Figure 5). While TBSA does affect RTW, so does which body region was primarily involved. It was found that those patients who had primary insult to the head and neck or upper limbs fared poorer in terms of RTW as compared to those who had a majority burn area over the chest and abdomen (Figure 6). The dataset also provided insights into the employment status of the burn victims. Self-employment, which included manual work/manual labor, constituted the most significant portion, followed by individuals employed in the service sector or business. Unemployed individuals also accounted for a notable proportion of the cases. RTW was, however, lower in the manual or unskilled labor group with less proportion of patients having RTW (Figure 7). The type of management received also affected RTW outcomes with those patients who were managed conservatively or had minor surgical procedures like small area split-thickness skin graft (STSG); patients who had less than two digits amputated had better RTW outcomes as compared to those who had major amputations or required massive area STSG or major flap reconstruction (Figure 8). From a total of 1130 patients, 710 (62.83%) patients returned to work, and 420 (37.16%) did not RTW.

## Discussion

Severely injured burn victims nowadays have an increased chance of survival because of major improvements in burn care. Patients with extensive burns are particularly prone to serious morbidity (e.g., scar formation, contractures, pain, psychological stress, and weakness), challenging returning to work and society. Our definition of RTW is "being able to perform activities of daily living independently, earning satisfactory employment and income (maintaining or increasing) and in the case of females (who were homemakers before suffering burns), continue to perform household activities in similar capabilities as before." Being able to RTW after suffering a burn injury is linked to normalcy and social integration, whereas not doing so has been linked to physical impairment and poor health-related quality of life. RTW is, therefore, one of the most crucial goals to pursue in burn recovery [1]. RTW has earned attention as a crucial result of burn treatment more recently. Work is an essential source of money for people and is also advantageous for many personal, social, and societal elements [2].

This cross-sectional data point is the most comprehensive information compiled from several investigations. It does not, and cannot, reveal the slope at which this process occurs [3]. The study's key results were that most former patients returned to work (62.83%) at the end of two years post injury. A two-year survey of 1130 patients suffering from burn injuries and admitted to the hospital revealed some significant results. Out of the total of 1130 patients, 710 patients had RTW (62.83%), revealing that a major portion of more than one-third of patients (37.17%) had difficulty in either gaining employment or continuing to work in the same capacity as before.

Out of all patients, 61.89% of males had RTW while 65% of females had RTW after two years of burn injury. The current study reported that 301 male patients (38.11%) could not RTW due to significant burns, resulting in poor financial conditions and health. Similar study findings were reported by Ifland et al. and Palmu et al., where 23% and 27.9% of males, respectively, could not RTW due to burns [8,9]. A poor RTW status was reported among young adults in the age range between 18-25 years (56.7%) and 25-40 years (40.0%), which can be attributed to lack of gain of employment, no established status of work at the time of burn injury, and incidences of psychological issues. The prevalence of psychological disorders was majorly observed in burn patients after six months, which was reported by Palmu et al. and their study also reported anxiety disorders (23%) and substance-related disorders (19%) in study participants [9]. This can also be attributed to our study, which observed almost 56.7% of young patients (less than 25 years of age) with no RTW status. However, we have not measured the exact relationship in all the age groups for no RTW status. When these data were analyzed in terms of gender-specific age groups, it was observed that young males who were less than 25 years old had poor RTW status (67.05%) explained by the facts that young males are involved in more risky activities and regional patterns of burn injuries suggesting major proportion being electric burn injuries and leading to limb loss or functional impairment. Also, these people are usually unemployed at the time of burn injuries, so they fail to gain employment due to both physical and psychological morbidity that these patients suffer. On the other hand, the female age group of 25-40 years had poorer RTW outcomes (56.19%) when compared to other groups explained majorly by social and cultural influences of females being seen as being incapable of carrying out their duties whether she was a homemaker or was previously employed. This demands a special attention of not only asking to treat women in the same light as males but also the psychological impact it has on women who are homemakers as they are dependent on their husbands or families to provide for them often affecting their whole quality of life.

The study also indicates fewer cases exhibited higher TBSA percentages, revealing more extensive burn injuries. Burn injuries affecting more than 40% of TBSA are considered severe and often require specialized medical intervention and prolonged treatment. These severe burns can have significant physical, psychological, and functional implications for the individuals involved. A total of 55.21% or 127 out of 230 patients suffering 40-60% TBSA burns of the cohort had returned to work. In our study, many of the cases (339) reported TBSA percentages of less than 20%, with 233 having RTW and 106 cases resulting in no RTW. Additionally, there were 380 cases in the 20-40% TBSA range, with 236 RTW and 144 cases of no RTW. There were 230 cases in the 40-60% TBSA range, with 127 RTW and 107 cases of no RTW. Lastly, 181 cases reported TBSA percentages greater than 60%, with 118 cases RTW and 63 cases no RTW; this higher RTW in this group is explained by the fact that most of the patients with such high TBSA involvement do not survive, and those who did have good recovery enabling them to RTW. Thus, this dataset reveals that with higher TBSA involvement, the ability to RTW is affected poorly and requires a longer course of management. Brych et al. reported an RTW percentage of 70-100% at the last reported follow-up (eight to 34 months) [10]. The TBSA affected by burns provides insights into the severity of the injuries. Most burn cases reported TBSA percentages of less than 20% and 20-40%. This suggests that most burn injuries were relatively localized and affected a smaller portion of the body. Such burns are generally considered less severe and have a higher likelihood of successful recovery. However, this does not provide the complete picture in the case of electric contact burns (ECB), which however localized are deep burns and often involve the loss of a limb or part of a limb due to necrosis and eventually require amputation. The proportion of TBSA (%) is one major factor affecting the RTW status in patients. Our study observed that patients with low TBSA (20-40% and <20%) had less RTW. A total of 144 patients (37.9%) and 106 patients (31.3%) had no RTW due to deep burns. These deep burns are mostly seen in TBSA (%) between 20-40% and are caused by electrical and flame burns. However, patients with high TBSA (%) had better recovery and more RTW status, which does not deeply affect the functioning of patients. The study conducted by Wasiak et al. reported similar findings where patients with burns, including TBSA > 30%, had a better rate of improvement in performing simple abilities and hand function between three and 12 months of injury. In addition, the study also reported poor physical scores in patients with larger burns (TBSA > 10%) [11].

The type of burn has been postulated to be one of the major factors associated with no RTW status. The present study revealed that 65.1% of the patients with electrical burns could not RTW. The low status of RTW has been attributed to the severity of electrical burns and restricted movement in patients. The cross-sectional survey conducted by Noble et al. also reported that electrical burns have resulted in poor quality of life among patients, including limited ability to RTW [12]. In addition, Xie et al. also reported poor quality of life among patients with extensive burn status and limited movement ability. The low ability to RTW was generally due to the restricted limb movements and functioning after an extensive burn among patients. Further, the study concluded that patients with extensive burns are more likely to have a poor quality of life compared to that of the general population in the long term due to poor physical functioning and the development of psychological problems [13]. Among flame and scald burns, large TBSA involvement was a major factor influencing RTW, since these subsets of patients not only involve having a slow recovery but often undergo major surgical procedures.

As revealed by the dataset, the employment status of burn victims sheds light on the occupational backgrounds of individuals affected by burn injuries. The data indicate that most burn cases were self-employed in manual and technical occupations. This suggests that individuals working for themselves in labor-intensive or skilled technical roles are more susceptible to burn incidents. This could be attributed to exposure to hazardous conditions or inadequate safety measures in these professions as well as the injuries involving extremities and most of these patients had no RTW (52.55%). Following self-employment, individuals in the service sector accounted for a significant portion of the burn cases. This could be attributed to service-related occupations, which often involve direct contact with customers, handling equipment or machinery, and exposure to various risk factors. In research involving working and unemployed participants, those with a mental history were less likely to RTW following a burn [10]. During the study, unemployed people had lower RTW than those who were employed before injury [2].

Our understanding of employment and economic productivity after burn injuries is expanded because of the participants who were able to find jobs again after their burn injury, over a quarter reported lower productivity [14]. The lower productivity may be explained by the mental and physical changes related to burn injury healing that may restrict the capacity to work the same number of hours or with the same effectiveness. In the early stages of their recovery, burn survivors may also have lower stamina, which might take several years to improve [15]. According to research, people with more severe burns are less likely to return to their jobs, mainly because it takes longer to find new employment after losing it, especially during short follow-up periods. However, there is a logical reason for the divergence in these results [16].

At the follow-up, the severity of the burn and the type of management predicted both the time to RTW in those who returned and the proportion of those who did not return. The RTW rates reported in some studies found that 33% had not returned to work, on average 4.7 years postburn, suggesting that longer follow-up periods do not result in a greater RTW rate [1]. Previous studies on RTW included patients working before the burn; about one-third had not returned to work 12 months after the burn [17]. In our study, we observed around 37.16% of patients did not RTW, which is higher than previous studies done in the West and explained by a lack of specialized programs to address burn victims and their rehabilitation as well as a lack of awareness besides financial limitations of burn victims. Those patients who were managed conservatively or required minor surgeries or amputation of fewer than two digits had a better RTW outcome whereas those patients who underwent major surgeries whether it be major limb amputation, major flap, or large TBSA grafting were unable to RTW (65.51%) since the rehabilitation and recovery takes much longer, requires multiple surgeries and often incomplete as there is lack of proper outreach of specialized facilities for rehabilitation in a developing country like ours and lack of awareness about the pathway to recovery and rehabilitation. Surgical interventions in burn patients have been seen as a preventive measure for mortality and morbidity and improving quality of life. However, conservative management revealed no RTW status in 32.1% (218 patients) and surgical management in 44.70% (202 patients). This can be due to the complex surgical procedure resulting in prolonged hospital stays, rehabilitation days, and days absent from work. Tang et al. assessed the factors resulting in no RTW, including surgical intervention, rehabilitation days, and quality of life among patients. Further, the study also reported that prolonged surgical interventions may increase hospital stays [18].

Overall, a detailed analysis of the employment status, TBSA percentages, and causes of burn injuries provide valuable insights into the factors contributing to burn incidents and RTW post-rehabilitation. This information can be utilized to develop targeted prevention strategies, improve occupational safety measures, and enhance public awareness regarding burn injury risks and precautions. This study can help bring a change in the approach to the management of burn victims broadening the horizon of burn care to focus not only on the injuries but on rehabilitation and the psychosocial impact that these injuries carry with them. As such helping these patients not only gain employment and RTW but also helps in their social reintegration with better mental and physical health. We also acknowledge that this study only acts as a guide to find areas of study in burn injury and more detailed and prospective studies. However, we do recommend further studies regarding RTW. Some guidelines are following a strict standardized treatment across burn centers, setting up more burn centers, early rehabilitation, and integration of psychiatric support in patient care. Also, the result of economic reintegration needs to be standardized, including income and quality of life adjusted to per capita income according to patient demographics.

## Conclusions

The data on burn injuries provide us with a wealth of information, shedding light on the various gender disparities, age group prevalence, burn types, discharge outcomes, causes, employment status, and TBSA involved. Moreover, the data show that the incidence of RTW can depend on various factors, such as the type of burn, cause, and affected TBSA. In our study, it was found that 62.83% of patients had a positive RTW status, while 37.17% did not have any RTW status. By analyzing these data, we can gain a better understanding of the factors contributing to burn injuries, and accordingly, develop targeted interventions aimed at reducing their occurrence. Additionally, we can use this information to ensure that burn victims receive the appropriate rehabilitation and support needed to reintegrate into society. Therefore, we must establish guidelines to ensure that burn victims can RTW, as this will aid in their recovery and contribute to their overall well-being.
